# Aggregation and neurotoxicity of recombinant α-synuclein aggregates initiated by dimerization

**DOI:** 10.1186/1750-1326-8-5

**Published:** 2013-01-22

**Authors:** Alireza Roostaee, Simon Beaudoin, Antanas Staskevicius, Xavier Roucou

**Affiliations:** 1 Department of Biochemistry, Université de Sherbrooke, 3001 12eme avenue nord, Sherbrooke J1H 5N4QC, Canada

## Abstract

**Background:**

Aggregation of the α-Synuclein (α-Syn) protein, amyloid fibril formation and progressive neurodegeneration are the neuropathological hallmarks of Parkinson's Disease (PD). However, a detailed mechanism of α-Syn aggregation/fibrillogenesis and the exact nature of toxic oligomeric species produced during amyloid formation process are still unknown.

**Results:**

In this study, the rates of α-Syn aggregation were compared for the recombinant wild-type (WT) α-Syn and a structurally relevant chimeric homologous protein containing an inducible Fv dimerizing domain (α-Syn^Fv^), capable to form dimers in the presence of a divalent ligand (AP20187). In the presence of AP20187, we report a rapid random coil into β-sheet conformational transformation of α-Syn^Fv^ within 24 h, whereas WT α-Syn showed 24 h delay to achieve β-sheet structure after 48 h. Fluorescence ANS and ThT binding experiments demonstrate an accelerated oligomer/amyloid formation of dimerized α-Syn^Fv^, compared to the slower oligomerization and amyloidogenesis of WT α-Syn or α-Syn^Fv^ without dimerizer AP20187. Both α-Syn^Fv^ and α-Syn pre-fibrillar aggregates internalized cells and induced neurotoxicity when injected into the hippocampus of wild-type mice. These recombinant toxic aggregates further converted into non-toxic amyloids which were successfully amplified by protein misfolding cyclic amplification method, providing the first evidence for the *in vitro* propagation of synthetic α-Syn aggregates.

**Conclusions:**

Together, we show that dimerization is important for α-Syn conformational transition and aggregation. In addition, α-Syn dimerization can accelerate the formation of neurotoxic aggregates and amyloid fibrils which can be amplified *in vitro*. A detailed characterization of the mechanism of α-Syn aggregation/amyloidogenesis and toxicity is crucial to comprehend Parkinson's disease pathology at the molecular level.

## Background

Parkinson's Disease (PD) is an age-related deterioration of dopaminergic neurons in the substantia nigra and other brain regions. The pathological hallmark of PD is the cytoplasmic deposition of amyloid-like aggregates termed Lewy Bodies , the fibrous inclusions which contain the protein α-Synuclein (α-Syn) [1-3]. α-Syn is a soluble, natively unfolded protein containing 140 a.a. which is expressed in the central nervous system including the cerebral cortex, hippocampus, amygdala, and olfactory bulb [4,5]. Although it is known that α-Syn is concentrated in presynaptic nerve terminals and appears to be associated with vesicular structures, its exact biological function is still uncertain [6,7]. Overexpression of the wild-type α-Syn or mutant variants (A30P, A53T, E46K) in several model organisms results in cytoplasmic inclusion formation followed by neurodegeneration [8,9].

PD belongs to a larger category of neurodegenerative diseases called protein misfolding disorders. The diagnostic feature common to protein misfolding disorders involves the deposition of insoluble protein aggregates and amyloid fibrils caused by accumulation of homologous proteins which are abnormally folded into β-sheet structures [10,11]. There is growing evidence that the pathogenic species causing cellular toxicity and neurodegeneration are non-fibrillar dimers and oligomeric assemblies [12-15]. Expression of α-Syn soluble oligomers with reduced fibrillization propensity induced neurotoxicity in worms, flies and mammalian neurons [16]. These pre-fibrillar aggregate species are readily formed *in vitro* from α-Syn mutants such as A30P and A53T, which are associated with neurotoxicity and early-onset PD [17,18]. In Alzheimer’s disease and transmissible spongiform encephalopathies, cytotoxicity of Amyloid-β oligomers and prion protein, respectively, is significantly more pronounced for rapidly formed pre-fibrillar aggregates than for highly-organized amyloid structures [19,20]. The exact biological function of amyloid inclusions is still under debate. Recent experimental data indicate the capacity of α-Syn amyloids to transfer between cells, both in cultured cells and in transgenic mice [21]. Interaction of α-Syn fibrils either with recombinant or cell-expressed α-Syn converts soluble homologous proteins into a misfolded state through a nucleation-dependent polymerization process [22,23]. These results suggest that the mechanism underlying propagation of α-Syn fibrils is reminiscent of those by which tau aggregates and prions spread and transmit the pathology through the brain tissue in Alzheimer’s and prion diseases, respectively [24].

The molecular mechanism underlying α-Syn transformation from its soluble monomeric form to the disease-associated oligomeric conformation has been a subject of intense research. Homodimer formation has been characterized as an essential factor in the aggregation pathway of numerous proteins, such as serpin, tau, and prion protein which are all involved in protein misfolding disorders [14,25,26]. In this regard, *in vitro* experiments have reported the existence of a dimeric state during conversion of α-Syn from soluble monomers to oligomeric species [27]. The dimerization of α-Syn induced by intramolecular oxidative cross-linking of tyrosine residues has been reported to induce the aggregation process [28]. Substitution of cysteine for tyrosine residues has been shown to induce α-Syn aggregation and in vitro toxicity [29]. Interaction of α-Syn with membrane phospholipids which results in the formation of β-sheet dimers and oligomers is considered to be a potential cytotoxic event, leading to the permeabilization of the cell membrane [30]. However, the importance of dimerization in α-Syn aggregation has not yet been directly tested. In addition, it is not known whether or not recombinant α-Syn dimers or oligomers, which are highly potent to transform into amyloid fibrils, are neurotoxic *in vivo*.

Here, we directly tested the hypothesis that dimerization of α-Syn is a key molecular step in the aggregation of α-Syn. We engineered a fusion protein between α-Syn and an FK506 binding domain (Fv). In the presence of AP20187, a divalent ligand that binds Fv, two protein molecules containing an Fv module are forced to interact and to dimerize. This strategy allows a fine regulation of induced dimeric interactions between Fv-containing proteins [14,31-34]. We demonstrate that dimerization is the central mechanism of aggregation and fibrillogenesis of α-Syn. Recombinant dimerization-induced oligomeric species, but not structured insoluble amyloids, were neurotoxic when injected to the brains of mice, consistent with the toxicity of cell-expressed α-Syn mutants with the propensity to form intermediate aggregates [16]. The α-Syn oligomers proceeded to form stable amyloids which could be amplified *in vitro* through the protein misfolding cyclic amplification (PMCA) method [35]. These results suggest that dimerization initiates α-Syn aggregation and provide direct evidence for the *in vivo* toxicity of α-Syn soluble prefibrillar aggregates.

## Results

### Dimerization accelerates α-Syn aggregation and amyloidogenesis

**Figure 1:**
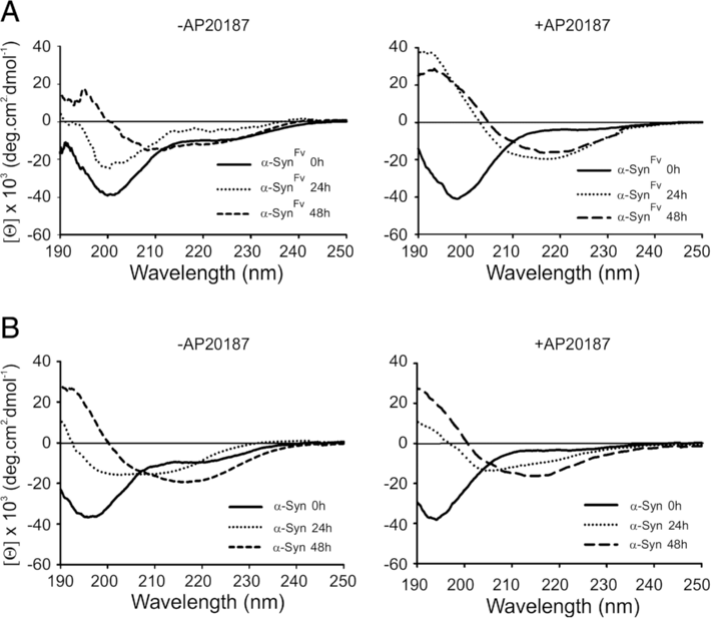
**Circular dichroic spectra of α-Syn**^**Fv **^**and α-Syn.** (**A**) α-Syn^Fv^ was incubated at different times in the absence (-AP20187) or in the presence (+AP20187) of dimerizer at 37°C with agitation. (**B**) α-Syn was incubated different times as indicated in (**A**). Both α-Syn^Fv^ and α-Syn displayed structural transition from random coil to β-sheet conformation.

Based on an inducible dimerizing system [14], we aimed to characterize the role of dimerization in α-Syn aggregation and fibrillogenesis. An Fv dimerizing domain was fused to the C-terminal of α-Syn, thereby creating α-Syn^Fv^ with the capacity to homodimerize in the presence of AP20187. Analysis of the circular dichroism spectrum of freshly purified recombinant α-Syn^Fv^ or α-Syn in the absence of AP20187 reveals a random coil structure with minimum molar ellipticity at 198 nm (Additional file 1: Figure S1, Figure 1A), consistent with α-Syn native secondary structure [36]. Both α-Syn^Fv^ and α-Syn showed a conformational transition from random coil to β-Sheet with a minimum at 216 nm within 48 h (Figure 1A,B) [36]. However, in the presence of AP20187, this transition was accelerated and occurred within 24 h. The secondary structure transformation of α-Syn was characterized by a negative peak with minimum ellipticity at 208 nm after 24h incubation (Figure 1B), consistent with recent demonstration of the existence of helical intermediates during α-Syn aggregation [37].

**Figure 2:**
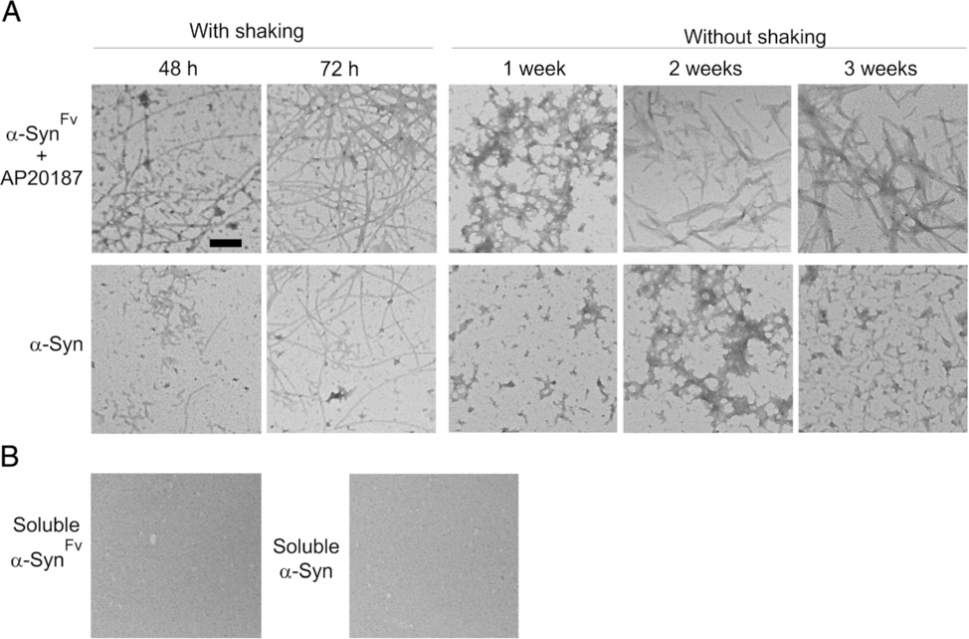
**Electron micrographs of α-Syn**^**Fv **^**and α-Syn protein preparations.** (**A**) α-Syn and α-Syn^Fv^ were incubated at 37°C in different conditions as indicated. (**B**) Freshly purified soluble α-Syn and α-Syn^Fv^ observed by electron microscopy. Scale bar, 100 nm.

**Figure 3:**
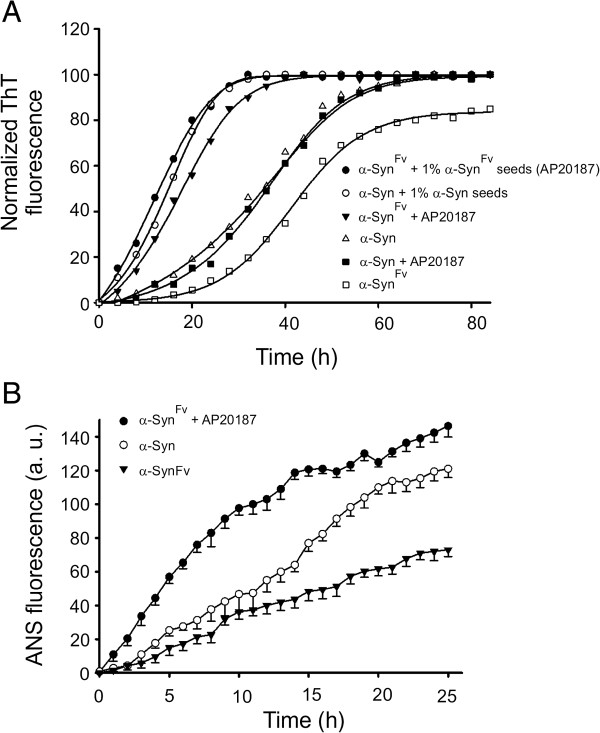
**Biophysical characterization of the aggregation of α-Syn proteins monitored by ThT and ANS fluorescence.** (**A**) Amyloidogenesis of α-Syn^Fv^ and α-Syn incubated at 37°C with agitation in different conditions was monitored with ThT over 80 h incubation time. (**B**) Exposure of hydrophobic regions was monitored with ANS during 0–24 h incubation time.

Soluble recombinant α-Syn converts into aggregated amyloids after 2–3 days of incubation with agitation at 37°C [36]; however, under static conditions, a prolonged incubation time (> 30 days) is needed for α-Syn to assemble into amyloid fibrils [17]. Thus, we examined whether induced α-Syn dimerization could increase the rate of amyloidogenesis under static or dynamic conditions. Electron micrographs of α-Syn preparations showed amyloid fibril formation in both stationary and agitating incubations with no detectable difference between the morphology of α-Syn^Fv^ or α-Syn fibrils under any experimental condition (Figure 2). Amyloid fibrils were clearly detected after dimerization of α-Syn^Fv^ following 48 h of agitation or two weeks without agitation. However, very few α-Syn fibrils were detected after 48 h of agitation, while under static conditions, α-Syn required 3 weeks incubation to form detectable fibrils (Figure 2). The kinetics of amyloid formation was then monitored by Thioflavin T (ThT) fluorescence. ThT exhibits a characteristic increase in fluorescence emission upon interaction with ordered β-sheet structures [38]. In the incubations including α-Syn^Fv^ plus AP20187, maximum fluorescence intensity was observed within 30h. Fibril quantification of α-Syn, in the presence or absence of AP20187, showed a longer lag phase and highest ThT emission achieved after 60h (Figure 3A). When 1% α-Syn aggregates or α-Syn^Fv^ dimerized aggregates were incubated with soluble α-Syn and α-Syn^Fv^, respectively, both proteins displayed the same capacity to trigger fibril formation (Figure 3A). However, α-Syn^Fv^ had a lower efficiency to polymerize into fibrils in the absence of dimerizing conditions (Figure 3A). These results also exclude any possibility that AP20187 or Fv domain could increase the rate of fibril formation by α-Syn constructs.

Kinetics of early aggregation of α-Syn was determined by the change in 8-anilino-1-naphthalenesulfonate (ANS) fluorescence intensity, due to the presence of a higher number of exposed hydrophobic sites, a key characteristic of early aggregates and oligomeric intermediate species [39]. ANS-binding experiments revealed the rapid formation of α-Syn^Fv^ pre-fibrillar aggregates within 2h of dimerization induced by AP20187, whereas assembly of α-Syn monomers into oligomers was associated with a significant (>2h) delay (Figure 3B).

### Accelerated formation of neurotoxic oligomers induced by α-Syn dimerization

The demonstration that dimerized α-Syn^Fv^ displayed a higher capacity for ANS-binding over 24 h of aggregation compared to α-Syn (Figure 3B) suggested that dimerization could accelerate the formation of toxic oligomeric species [40]. To verify this hypothesis, neurotoxicity of aggregates and amyloid fibrils formed by α-Syn or α-Syn^Fv^ was determined by subcortical inoculation of proteins into the right hippocampus of wild-type C57BL/6 mice [41]. Indeed, hippocampal neurons are highly susceptible to the neurodegeneration caused by α-Syn aggregation [42].

**Figure 4:**
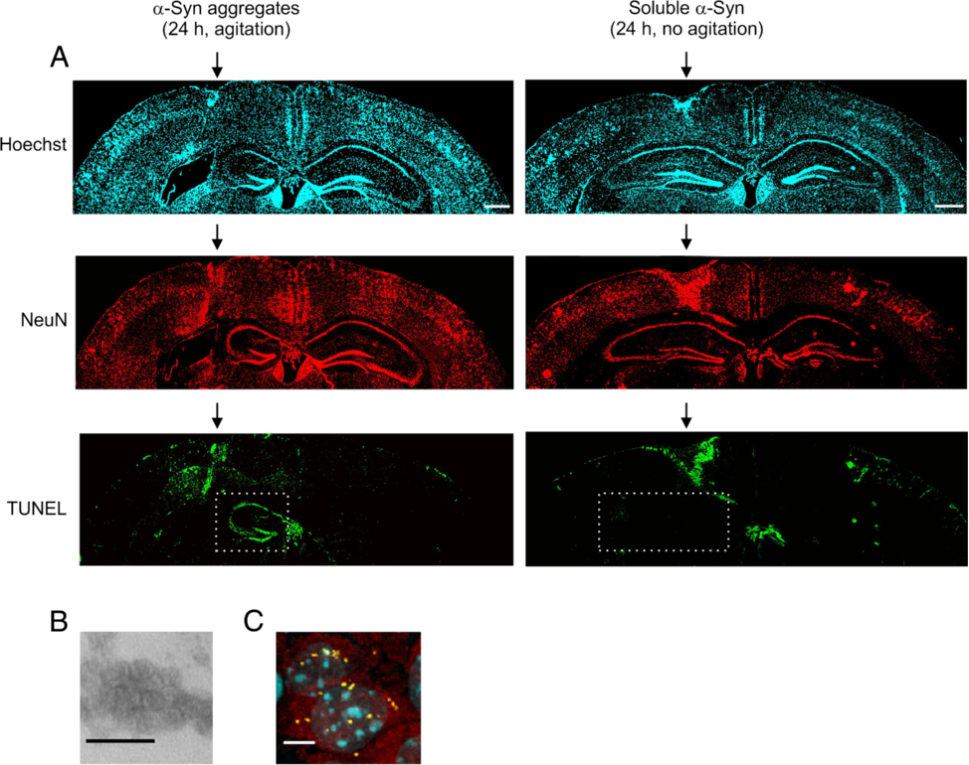
***In vivo *neurotoxicity of synthetic α-Syn oligomers.** (**A**) α-Syn protein was treated as indicated and injected into the brain of wild-type C57BL/6 mice. Apoptotic neurons of the hippocampal region adjacent to the injection site were detected by TUNEL assay (green channel) followed by confocal microscopy. Hippocampus in the injected hemisphere is shown in a dotted box. The neuronal marker NeuN is detected by immunofluorescence (red channel). Nuclei are stained with Hoechst (blue channel). Scale bar, 500 μm. (**B**) Electron micrographs of toxic α-Syn oligomers obtained after incubation at 37°C for 24 h. Scale bar, 25 nm. (**C**) Synthetic α-Syn aggregates were labeled with Alexa Fluor 633 prior to injection and detected by confocal microscopy (aggregates, yellow channel; NeuN, red channel; nucleus, blue channel). Scale bar, 10 μm.

The susceptibility of mice to α-Syn-induced neurotoxicity was determined by monitoring hippocampal cells for apoptosis 48 h post-injection by terminal deoxynucleotidyl transferase dUTP nick end labeling (TUNEL) assay. Neuronal cells were identified by immunofluorescence with an antibody against the neuronal marker NeuN. Formation of toxic aggregates was highly dependent on the oligomerization state of α-Syn. Following agitating incubations, both dimerized α-Syn^Fv^ (Additional file 2: Figure S2) and α-Syn (Figure 4) transformed into highly neurotoxic oligomers within 10 h and 24 h, respectively, and caused severe apoptosis in hippocampal neurons (Figure 4A, Additional file 2: Figure S2A). The protein toxic species were morphologically higher-ordered aggregates of heterogeneous size, such as annular and amorphous protofibrillar oligomers (Figure 4B, Additional file 2: Figure S2B), with a high capacity to bind ANS (Figure 3B). In contrast to the oligomers, neither soluble α-Syn or α-Syn^Fv^ proteins nor insoluble amyloid fibrils were neurotoxic in our experiments (Figure 4A, Additional file 2: Figure S2A, Additional file 3: Figure S3A). AlexaFluor 633-labeled oligomers and amyloid fibrils were clearly visible in the cytoplasm of neurons (Figure 4B, Additional file 2: Figure S2C,D Additional file 3: Figure S3B). Therefore, dimerization of α-Syn enhances the formation rate of toxic oligomers which can further transform into insoluble amyloid fibrils lacking neurotoxic activity.

### *In Vitro* amplification of α-Syn fibrils

**Figure 5:**
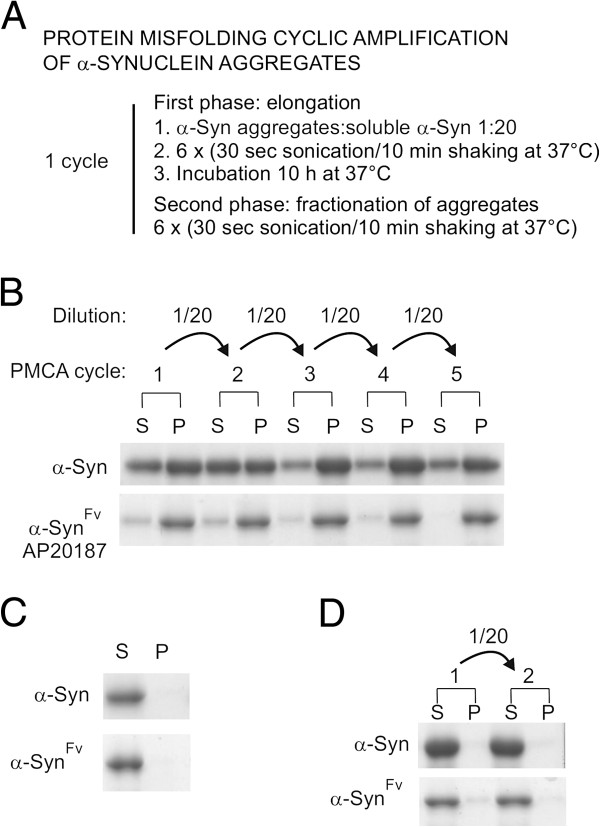
**PMCA reactions seeded with α-Syn or α-Syn**^**Fv **^**aggregates.** (**A**) Illustration of PMCA method used to amplify α-Syn or α-Syn^Fv^ aggregates in the presence of homologous soluble proteins (see Materials and Methods). (**B**) α-Syn aggregates or α-Syn^Fv^ aggregates induced by AP20187 were diluted 20 times into freshly prepared soluble proteins of the same species. Supernatant (S, soluble) and pellet (P, insoluble) fractions recovered from centrifugation of 3 μg protein samples withdrawn at the end of each PMCA cycle were analyzed by SDS-PAGE and Coomassie blue staining. This result is representative of three independent experiments. (**C**) α-Syn or α-Syn^Fv^ aggregates were diluted 1/20 into soluble homologous proteins and immediately centrifuged. The absence of detectable protein in the pellet fraction indicates the lack of amplification of aggregates. (**D**) α-Syn or α-Syn^Fv^ aggregates were submitted to two PMCA cycles with incubations performed under stationary conditions.

In PD pathology, topographical progression of α-Syn aggregated amyloids or Lewy Bodies includes stereotypical spread of Lewy Body formation from the lower brainstem and anterior olfactory structures into the limbic system, neocortex and other brain areas [43]. Recent data reported cell-to-cell transmission of α-Syn aggregates both in cultured cells and in transgenic mice [21]. These data strongly suggest that α-Syn aggregates can polymerize by recruitment of soluble homologous proteins and fragment to generate new seeds which retain the capacity to nucleate newly synthesized soluble monomers, thereby propagating protein aggregates through the nervous system, in a mechanism similar to prion propagation [23,44]. In order to determine if dimerized α-Syn^Fv^ aggregates could also display self-propagating activity, we generated a modified *in vitro* conversion assay similar to the PMCA protocol which has been used to convert cellular prion protein into infectious prion aggregates [35]. Using this assay we aimed to characterize the seeding effect of dimerization on the initiation of propagation of α-Syn^Fv^ aggregates in the conditions relevant to the *in vivo* spread of pathogenic form of α-Syn. Indeed, dimerization of α-Syn might lead to a prion-like propagation which ultimately may have implications for the spread of PD pathology *in vivo.* As a positive control, we used wild-type α-Syn aggregates which have been shown to be transmissible when injected to the mouse brain [45]. Each cycle of PMCA was composed of two phases (Figure 5A). During the first phase, α-Syn and α-Syn^Fv^ aggregates were diluted 20 times with soluble homologous protein and incubated 12 h with constant agitation to induce the formation of amyloids. This dilution factor was selected based on the observation that seeding with 5% aggregates resulted in maximum ThT binding within 12 h (Additional file 4: Figure S4A). Formation of large polymers in the samples incubated for 12 h was determined by centrifugation and SDS-PAGE analysis. The obtained results confirmed that both α-Syn and α-Syn^Fv^ were predominantly insoluble at the end of a 12 h polymerization phase (Additional file 4: Figure S4B). During the second phase of PMCA, α-Syn and α-Syn^Fv^ amyloids were sonicated in order to fractionate fibril polymers and multiply seeds for subsequent polymerization [46]. Centrifugation and analysis of proteins in supernatant and pellet fractions by SDS-PAGE confirmed the amplification of insoluble aggregates by PMCA (Figure 5B). Densitometric analysis of the proteins in three independent experiments definitively established that both α-Syn and α-Syn^Fv^ aggregates were efficiently amplified through PMCA using our protocol (Additional file 5: Figure S5). High molecular weight SDS-resistant species including dimers were formed during the PMCA and could be detected by western blot (Additional file 6: Figure S6A). The conversion of soluble α-Syn and α-Syn^Fv^ into aggregated states and higher molecular weight species was observed through at least five serial reactions (Figure 5B, Additional file 6: Figure S6A). In a parallel experiment, samples were immediately centrifuged after 1/20 dilution of sonicated aggregates instead of being submitted to a 12 h polymerization phase (Figure 5C). No significant levels of insoluble proteins used to seed the reaction were detected, indicating that insoluble fractions detected after the PMCA cycles were essentially formed from soluble monomers during the polymerization phase. In control assays where proteins were subjected to polymerization in a static phase for 12 h, no amplification of protein aggregates was recorded, indicating the requirement of agitation for an efficient conversion (Figure 5D). The dilution factor for the initial seeds used to start PMCA was estimated to be 3.2 × 10^-6^ in the fifth cycle. This concentration of seeds was insufficient to account for the conversion of soluble proteins in PMCA cycles, demonstrating that *de novo* generated α-Syn and α-Syn^Fv^ aggregates also gained their own seeding activity, as shown by agitation-induced conversion experiments (Figure 5B). In a control experiment, no formation of insoluble aggregates was detected in the absence of added aggregates to initiate the polymerization (Additional file 6: Figure S6B). This result indicates that sonication and agitation do not induce α-Syn aggregation in the absence of seeding aggregates.

## Discussion

A detailed characterization of α-Syn aggregation and amyloidogenesis is essential to comprehend the molecular mechanisms of neurotoxicity and pathology spread in PD. A critical challenge in understanding the mechanism of α-Syn pathology, and in the subsequent design of intervention strategies, has been the initiation of protein polymerization and the correlation of aggregate formation with biological effects, such as neurotoxicity and amyloid formation. In this study we describe the importance of dimerization in initiation and acceleration of α-Syn aggregation and neurotoxicity. Under physiological-like conditions, toxic aggregates can transform into non-toxic amyloid fibrils which are amplified *in vitro* in the presence of homologous soluble proteins, thereby generating *de novo* amyloids. The results discussed herein can open new insights into the mechanism of α-Syn oligomerization which is directly linked to the pathology of PD.

### Importance of dimerization in formation of α-Syn toxic aggregates and amyloid fibrils

Homodimerization of amyloid-forming proteins such as prion protein, amyloid-β and serpin has been characterized as a primary factor involved in aggregation and pathogenesis of prion encephalopathies, Alzheimer’s disease and serpinopathies, respectively [14,26,47]. Here, we extend these observations to α-Syn, as well, since α-Syn dimerization induces a conformational change to a β-sheet rich structure (Figure 1). This results in a considerable increase in the rate of α-Syn aggregation and amyloidogenesis (Figures 2 and 3A). In comparison to α-Syn, accelerated aggregation of α-Syn^Fv^ was associated with the increased binding of ANS (Figure 3B), suggesting that dimerization of α-Syn induced protein aggregation through increase in surface hydrophobicity within the amino acid side chains of aggregating proteins [40]. The influence of hydrophobic interfacial area was recently emphasized as a new factor for accelerating α-Syn aggregation and amyloidogenesis [48]. In agreement with the importance of dimerization for α-Syn aggregation and pathogenesis, another study has shown that dimeric intermediates of WT and PD-associated mutants of α-Syn were relevant species in protein aggregation and cellular toxicity [49,50]. The increased aggregation rate of dimerization-induced α-Syn may help misfolded proteins to evade the cellular clearance machinery. In this scenario, a feedback loop is generated through which α-Syn aggregates impair the proteasome-mediated protein degradation pathway [50], resulting in a further shift in the population of α-Syn from soluble into cellular aggregates and amyloid inclusions which are involved in PD pathogenesis.

Acceleration of protein aggregation and formation of prefibrillar oligomers have been proposed to be associated with PD-related neurotoxicity [16,18], however, there is a lack of direct *in vivo* evidence on the neurotoxicity of α-Syn oligomers and protofibrils. Previously, α-Syn oligomers were shown to be toxic in rat brains, but animals were not directly injected with recombinant oligomers; they were infected with lentiviruses expressing α-syn mutants (E57K and E35K) that favor oligomer assembly but lack the amyloid forming capacity [51]. It should be noted that α-Syn oligomerization is associated with amyloidogenesis in most of the clinical cases of Parkinson's disease. In this context, our results demonstrating that α-Syn oligomeric species formed during the amyloidogenesis process are neurotoxic species may be physiologically more relevant. In addition, dimerization of α-Syn can significantly accelerate the formation of toxic oligomeric species and amyloid fibrils with no toxic activity (Figure 4). Although toxic protein aggregates contained spherical or amorphous ultrastructures, the presence of smaller toxic species undetectable by electron microscopy cannot be discarded. Recent results on HypF-N, a bacterial protein not associated with any amyloid disease, indicate that hydrophobicity of oligomeric assemblies is a major determinant of membrane interaction and cytotoxicity of aggregate oligomers [40]. These observations are consistent with the theory that hydrophobicity of protofibril intermediates aids penetration of the membrane lipid bilayer in order to form pore-like structures which disrupt normal cellular physiology and cause neurotoxicity [52]. Further research is required to evaluate this proposal and other mechanistic issues concerning neurotoxicity, as well as the exact biological activity of α-Syn aggregates.

### PMCA of α-Syn aggregates

Emerging studies of protein misfolding disorders suggest that the molecular mechanism underlying transmission of protein aggregation in Alzheimer’s, Huntington’s and Parkinson’s diseases is similar to prion diseases [11,45,53,54]. Recently, it was shown that a single intrastriatal inoculation of synthetic α-Syn fibrils led to the cell-to-cell transmission of pathologic α-Syn and PD-like Lewy pathology in anatomically interconnected regions [45]. Based on these evidences, we decided to directly evaluate the *in vivo* theories on the propagation of α-Syn aggregates and amyloid fibrils by developing a modified PMCA assay (Figure 5A). In addition, we investigated whether α-Syn amyloids seeded by dimerization could initiate an *in vitro* cascade of nucleation/polymerization through PMCA cycles. Our results demonstrate that dimerization enhances the aggregation of α-Syn, and fragmentation of α-Syn aggregates can provide novel nucleation sites to induce conformational conversion of newly added α-Syn soluble monomers (Figure 5B). This *in vitro* propagation pattern of α-Syn proposes that only the interaction between aggregates and soluble α-Syn is sufficient to initiate amplification of α-Syn aggregates. Formation of α-Syn aggregates has been recently characterized *in vivo* in grafted human fetal mesencephalic neurons implanted in the brain of a patient with PD [53]. Therefore, our demonstration of the *in vitro* replication of α-Syn amyloid aggregates in the absence of cellular factors provides evidence for the existence of a self-propagation system by which Lewy Body formation eventually spreads throughout the nervous system. Further research will have to determine whether the PMCA intermediate products are neurotoxic and/or able to propagate after injection into the brain of animal models by inducing the aggregation of endogenously expressed α-Syn.

### Could α-Syn dimers be a therapeutic target?

The phenomenon of protein dimerization has been observed during aggregation and amyloidogenesis of α-Syn. The A30P and A53T mutants, which are linked to familial PD, display a faster rate of protein aggregation and show a great propensity to self-interact and form dimeric structures [17,28,49,50,55]. Here, we directly show that α-Syn dimerization accelerates formation of neurotoxic intermediate aggregate species and amyloid fibrils both of which are associated with PD pathology. Therefore, compounds that prevent or slow down the dimerization rate of α-Syn can block or hinder protein aggregation which otherwise would be followed by a rapid accumulation and deposition of neurotoxic oligomers and amyloid inclusions. This information could be used to evaluate transgenic animal models with accelerated PD pathology caused by α-Syn dimerization. However, extensive research is still required to indentify *in vivo* factors, such as uptake or release of α-Syn by cells, involved in cell-to-cell transfer of protein aggregates with seeding activity during pathology spread throughout the nervous system [56].

## Conclusions

By using a combination of *in vitro* and *in vivo* approaches, we propose a mechanistic link between α-Syn dimerization and aggregation with consequent neurotoxicity. Indeed, this study provides direct evidence on the impact of dimerization on the α-Syn oligomerization and amyloidogenesis rates. Acceleration of oligomerization can subsequently induce the formation of neurotoxic aggregates and amyloid fibrils which can be propagated *in vitro* in a prion-like manner. The important finding that both α-Syn and α-Syn^Fv^ synthetic aggregates can induce neurotoxicity confirms the literature data and provides new experimental tools for studying a direct cause and effect relationship between the aggregation of α-Syn and formation of neurotoxic species. Finding the precise correlation between different oligomeric structures and neurotoxicity in PD pathogenesis is essential to comprehend the molecular mechanism of pathology. In this context, characterization of the globular aggregates as neurotoxic species is an important step toward understanding the pathology of PD at the molecular level.

## Methods

### Cloning of α-Syn constructs

Total RNA was extracted from HeLa cells using the Trizol reagent (Invitrogen), according to manufacturer's instructions. α-Syn and α-Syn-Fv constructs were generated using PCR primers listed in Additional file 7: Table S1. α-Syn cDNA was produced using α-Syn-Fw and α-Syn-Rv PCR primers specific to the 3’ and 5’ untranslated region of α-Syn mRNA. The full-length C-terminally 6xHis-tagged recombinant α-Syn was amplified from α-Syn cDNA using the α-Syn-His-Fw and α-Syn-His-Rv primer pair. α-Syn cDNA was also used as a template to produce 6xHis-tagged recombinant α-Syn^Fv^ by overlap extension PCR, using α-Syn^Fv^-Rv, α-Syn^Fv^-Fw and Fv-His-Rv primers. Fv was amplified from pC4-Fv1E (Invitrogen). To avoid cysteine misincorporation at codon 136 in bacterially expressed α-Syn constructs and artefactual autodimerization [57], site-directed mutagenesis of codon 136 (Y136-TAC to Y136-TAT) was done using α-Syn-Fw^Y136-TAT^/ α-Syn-Rv^Y136-TAT^ and α-Syn-Fv-Fw^Y136-TAT^/α-Syn-Fv-Rv^Y136-TAT^ primer pairs to generate α-Syn and α-Syn^Fv^ final constructs, respectively. PCR amplification fidelity was confirmed by sequencing and constructs were inserted into PET-21b vectors using NdeI and HindIII cloning sites.

### Expression and purification of α-Syn and α-Syn^Fv^

Constructs were overexpressed in BL21(DE3)pLysS cells at 25°C with slow shaking, using 0.1 mM IPTG (isopropyl β-D-thiogalactoside) for 10 h. The bacterial pellets were lysed in 300 mM NaCl, 50 mM NaH_2_PO_4_ (pH 8.0), 1 mM PMSF, 0.1 mM TCEP [tris-(2-carboxyethyl)phosphine] and 1 mg/ml lysozyme, followed by either drawing–expelling of the lysates through a 21-gauge-needle or a brief sonication followed by centrifugation for 45 min at 20000 *g*. Purification of the protein from supernatant was carried out using nickel-affinity chromatography according to the manufacturer's protocol (Qiagen). Proteins were eluted in a buffer consisting of 125 mM NaCl, 300 mM imidazole, 0.1 mM TCEP, 25 mM NaH_2_PO_4_ (pH 7.4). This buffer was then exchanged for a physiological buffer [58] consisting of 50 mM K_3_PO_4_, 2 mM KCl, 70 mM CH_3_COOH, 9 mM KH_3_PO_4_ and 5 mM Na_2_HPO_4_ (pH 7.4), using Millipore ultracentrifugation filters (Amicon Canada). The concentration of proteins was estimated by the absorbance at 280 nm, considering molar absorption coefficients of 15392 M^−1^·cm^−1^ and 28660 M^−1^·cm^−1^ for 6xHis-tagged α-Syn and α-Syn^Fv^, respectively. The recombinant proteins were detected by SDS-PAGE and western blot using monoclonal antibody (EP1646Y) to α-Syn.

### Amyloid assembly experiments

α-Syn^Fv^ (5.7 mg/ml) and α-Syn (3.0 mg/ml) in the presence or absence of 10 μM AP20187 (divalent ligand) or 10 μM FK506 (monovalent ligand) were used in the amyloid assembly preparations. Samples were incubated in 0,5 ml physiological buffer supplemented with 0,02% NaN_3_ (pH 7.4) in microtubes with or without agitation at 300 rpm at 37°C. Aliquots were withdrawn at different time intervals from samples incubated in agitation or static conditions, and were analyzed by biochemical and biophysical methods (see below).

### Circular dichroism spectroscopy

Circular dichroism measurements were done as described before [59]. α-Syn^Fv^ and α-Syn were taken from amyloid assembly incubations and diluted to 20 μM in phosphate buffer (25 μM Na_2_HPO_4_). Samples were transferred into 0.1 cm light-path quartz cuvettes and far UV spectra (190–250 nm) were recorded on a Jasco J-810 CD spectrophotometer at 25°C. Three consecutive scans were accumulated and the average spectra were recorded. Raw data were converted to molar ellipticity and plotted as a function of wavelength.

### Thioflavin T and 8-anilino-1-naphthalenesulfonate binding

Thioflavin T binding to α-Syn and α-Syn-Fv amyloid samples withdrawn from amyloid assembly experiments at the indicated time points was evaluated as described before. Binding of 8-anilino-1-naphthalenesulfonate to α-Syn and α-Syn^Fv^ was evaluated by measuring the fluorescence enhancement of 8-anilino-1-naphthalenesulfonate (50 μM) in the presence of 85 μM protein preparation upon excitation at a wavelength of 380 nm. The emission spectra were integrated from 400 to 600 nm. Values are expressed as the mean ± S.E.M. of maximum fluorescence intensity from three independent experiments.

### Electron microscopy

A 10 μL sample from amyloid assembly preparations was taken for EM analysis. Samples included α-Syn^Fv^ or α-Syn amyloids after 10 h, 24 h and 48 h agitation at 37°C. Aliquots were also withdrawn after 1, 2 and 3 weeks from stationary incubations. Protein samples were applied to formar- and carbon-coated grids. The excess fluid was drained with filter paper and the samples were stained for 1 min with 2% uranyl acetate. The grid was air-dried and examined in a Hitachi H-2500 electron microscope at 80 kV at a magnification of 15–75000.

### *In vivo* neurotoxicity experiments

Inocula were protein aliquots prepared with sterile solutions withdrawn from amyloid assembly experiments after 10 h, 24 h, and 48 h shaking at 37°C. One month old male WT C57BL/6 mice were anesthesized with ketamine/xylazine (1ml/kg, 87/13) and positioned on a stereotaxic frame. Coordinates for the injection site were 1.50 mm posterior and 1.75 mm ventral to the bregma and 2.00 mm lateral to the midline. 2 μl physiological buffer or 2 μl protein samples (40 μM) were injected at a rate of 0.4 μl/min. After 48 h, mice were anesthetized using isoflurane followed by euthanasia by cervical dislocation, according to the Animal Ethics Committee of the Université de Sherbrooke. A group of 18 mice was injected. Each of the three protein preparations **(**protein aliquots withdrawn from amyloid assembly experiments after 10 h, 24 h, and 48 h shaking at 37°C for α-Syn^Fv^; and protein aliquots withdrawn from amyloid assembly experiments after 24 h, 48 h, and 72 h shaking at 37°C for α-Syn) was injected in three mice. Two independent experiments were performed, with a total of 36 mice.

### Detection of apoptotic cells

To detect apoptotic cells, brain sections adjacent to the injection site were tested by terminal deoxynucleotidyl transferase dUTP nick end labeling (TUNEL) assay using Dead End Fluorometric TUNEL System (Promega), according to the manufacturer’s protocol. Nuclei were also stained with Hoechst. For confocal analysis, brain sections were examined with a scanning confocal microscope (FV1000, Olympus, Tokyo, Japan) coupled to an inverted microscope with a 63X oil immersion objective (Olympus). Specimens were laser-excited at 488 nm (TUNEL-positive signals) and 405 nm (Hoechst). In order to avoid cross-talk between the fluorescence emitted by Hoechst (405) and Alexa 488, images were acquired sequentially. Serial horizontal optical sections of 320 X 320 pixels were taken at 1.0 μm intervals through the entire thickness of the tissue (optical resolution: lateral 0.18 μm; axial 0.25 μm). Identical settings of the instrument were used for all photos. For illustration purposes, images were pseudocolored according to their original fluorochromes, merged (FluoView software, Olympus), then cropped and assembled (Adobe Photoshop software, Adobe Systems, Mountain View, CA).

### Detection of recombinant α-syn species in neuronal cells

Protein preparations were labeled with Alexa Fluor 633 a protein labeling kit as described by the manufacturer (Molecular Probes, A20170). The Alexa 633-labeled inocula were injected in the mice brain as described. Brain nuclei were stained with Hoechst and neuronal cells were identified by immunofluorescence using NeuN antibody. Brain sections were examined with a scanning confocal microscope with a 60X oil immersion objective (Olympus). Specimens were laser-excited at 633 nm to detect Alexa-labeled α-syn and α-Syn^Fv^ preparations, 568 nm (NeuN) and 405 nm (Hoechst). In order to avoid cross-talk between the fluorescence emitted by different dyes, images were acquired sequentially. Serial horizontal optical sections of 320 X 320 pixels were taken from CA2-CA3 hyppocampal neurons and focal planes were stacked for optimal α-syn detection. Identical settings of the instrument were used for all photos. For illustration purposes, each dye was pseudocolored (FluoView software, Olympus).

### Protein misfolding cyclic amplification (PMCA) of α-Syn

PMCA reactions were prepared in 1.7 ml ultraclear microtubes as 300 μl solutions containing physiological buffer (pH 7.4). In the first cycle, 22.5 μl aliquots from 48 h fibril assembly experiments of α-Syn^Fv^ or α-Syn were added to 300 μM soluble α-Syn^Fv^ and α-Syn in physiological buffer solutions, respectively, to give a fibril/protein ratio of 1:20. In the elongation step, reactions were initiated by incubating samples at 37°C with shaking (300 rpm) for 1 h during which 6X periodic sonication/incubation cycles (30s sonication followed by 10 min shaking) were performed to prepare homogeneous aggregate seeds. This step was followed by incubation of samples for 10 h with constant shaking (37°C) to elongate amyloid fibrils. After 10 h incubation, newly formed amyloids were subjected to periodic sonication/incubation cycles for 1 h (as described above) to fractionate amyloid fibrils in order to provide a solution of amyloid seeds. Fractionated amyloids were then diluted 1/20 in physiological buffer containing 300 μM soluble proteins and identical elongation and fractionation cycles were applied to complete the PMCA round. These cycles were consecutively repeated at least five times and at the end of each PMCA round, 50 μl aliquots were withdrawn centrifuged at 20 000 *g* for 1 h to separate insoluble amyloids from the supernatant. Proteins from the supernatant fractions were precipitated with four volumes of methanol. 2 μg of protein from each pellet or supernatant were subjected to SDS-PAGE analysis and Coomassie blue staining and band densities were analyzed with AlphaEaseFC software (Alpha Innotech). To verify the importance of shaking in the efficiency of PMCA, control cycles were performed in static conditions. To confirm the importance of elongation, after 1/20 dilution of amyloid seeds, samples were spun as described and both soluble and insoluble fractions were stored at −20°C until SDS-PAGE analysis.

## Abbreviations

α-Syn: α-Synuclein; Fv: FK506 binding domain; PD: Parkinson’s disease; PMCA: Protein Misfolding Cyclic Amplification.

## Competing interests

The author declares that they have no competing interests.

## Authors’ contributions

AR, SB, and AS performed the experiments. AR and XR designed the study and wrote the manuscript. All authors read and approved the final manuscript.

## Supplementary Material

Additional file 1: Figure S1 Expression and purification of α-Syn and α-Syn^Fv^. Recombinant proteins were produced in BL21(DE3)pLysS *Escherichia coli* cells, as described under “Materials and Methods”. 2 μg of recombinant proteins was subjected to the SDS-PAGE and Coomassie blue staining (left panel). Purity of the proteins was estimated to be superior to 95%. Identity of the proteins was confirmed by immunoblotting using monoclonal anti-α-Syn EP1646Y antibody (right panel). Click here for file

Additional file 2: Figure S2 *In vivo* neurotoxicity of synthetic α-Syn^Fv^ oligomers. (*A*) α-Syn^Fv^ protein was treated in the absence (soluble) or in the presence of 10 μM AP20187 (aggregates and amyloids) as indicated, and injected into the brain of wild-type C57BL/6 mice. Apoptotic neurons of the hippocampal region adjacent to the injection site were detected by TUNEL assay (green channel) followed by confocal microscopy. Hippocampus in the injected hemisphere is shown in a dotted box. The neuronal marker NeuN is detected by immunofluorescence (red channel). Nuclei are stained with Hoechst (blue channel). Scale bar, 500 μm. (*B*) Electron micrographs of toxic α-Syn^Fv^ oligomers obtained after incubation at 37 ºC for 10 h. Scale bar, 25 nm. (*C*) Synthetic α-Syn^Fv^ aggregates were labeled with Alexa Fluor 633 and detected by confocal microscopy (aggregates, yellow channel; NeuN, red channel; Nucleus, Blue channel). Scale bar, 10 μm. (*D*) Synthetic α-Syn^Fv^ amyloids were labeled with Alexa Fluor 633 and detected by confocal microscopy (aggregates, yellow channel; NeuN, red channel; nucleus, blue channel). Scale bar, 10 μm. Click here for file

Additional file 3: Figure S3 Absence of *in vivo* neurotoxicity of synthetic α-Syn amyloids. (*A*) α-Syn protein was treated as indicated, and injected into the brain of wild-type C57BL/6 mice. Apoptotic neurons of the hippocampal region adjacent to the injection site were detected by TUNEL assay (green channel) followed by confocal microscopy. Hippocampus in the injected hemisphere is shown in a dashed box. The neuronal marker NeuN is detected by immunofluorescence (red channel). Nuclei are stained with Hoechst (blue channel). Scale bar, 500 μm. (*B*) Synthetic α-Syn amyloids were labeled with Alexa Fluor 633 and detected by confocal microscopy (aggregates, yellow channel; NeuN, red channel; nucleus, blue channel). Scale bar, 10 μm. Click here for file

Additional file 4: Figure S4 Acceleration of α-Syn amyloid formation in seeding incubations. (*A*) 200 μM of freshly prepared recombinant α-Syn or α-Syn^Fv^ was incubated with 2% or 5% homologous amyloid seeds with agitation at 37°C for 80 h. α-Syn^Fv^ seeds were produced in the presence of AP20187. α-Syn seeds were obtained by agitating α-Syn for 48 h at 37ºC. (*B*) Aliquots withdrawn at 4 h intervals from the incubations including 5% seed were analyzed by centrifugation, SDS-PAGE and Coomassie blue staining. Click here for file

Additional file 5: Figure S5 Densitometric analysis of PMCA reactions with α-Syn and α-Syn^Fv^ in three independent experiments. α-Syn aggregates or α-Syn^Fv^ aggregates induced by AP20187 were diluted 20 times into freshly prepared soluble proteins of the same species. Supernatant (S, soluble) and pellet (P, insoluble) fractions recovered from centrifugation of 3 μg protein samples withdrawn at the end of each PMCA cycle were analyzed by SDS-PAGE and Coomassie blue staining. Click here for file

Additional file 6: Figure S6 Analyses of PMCA reactions. (*A*) SDS-resistant α-Syn and α-Syn^Fv^ species after PMCA in pellet fractions. At the end of each PMCA cycles using purified recombinant proteins (Figure Figure 5), proteins in the pellet were analyzed by western blot using monoclonal anti-α-Syn EP1646Y antibody. (*B*) Control experiments in the absence of seeds. The PMCA reaction was not seeded with α-Syn^Fv^ aggregates. At the end of each PMCA cycle, proteins in the pellet were analyzed by western blot using monoclonal anti-α-Syn EP1646Y antibody. Click here for file

Additional file 7: Table S1 Oligonucleotides used to produce α α-Syn constructs. Click here for file
