# Supplementary figures and images for: Aggregation and neurotoxicity of recombinant α-synuclein aggregates initiated by dimerization
Source: Mol Neurodegener. 2013 Jan 22;8:5. doi: 10.1186/1750-1326-8-5 (PMC3764494; doi:10.1186/1750-1326-8-5)

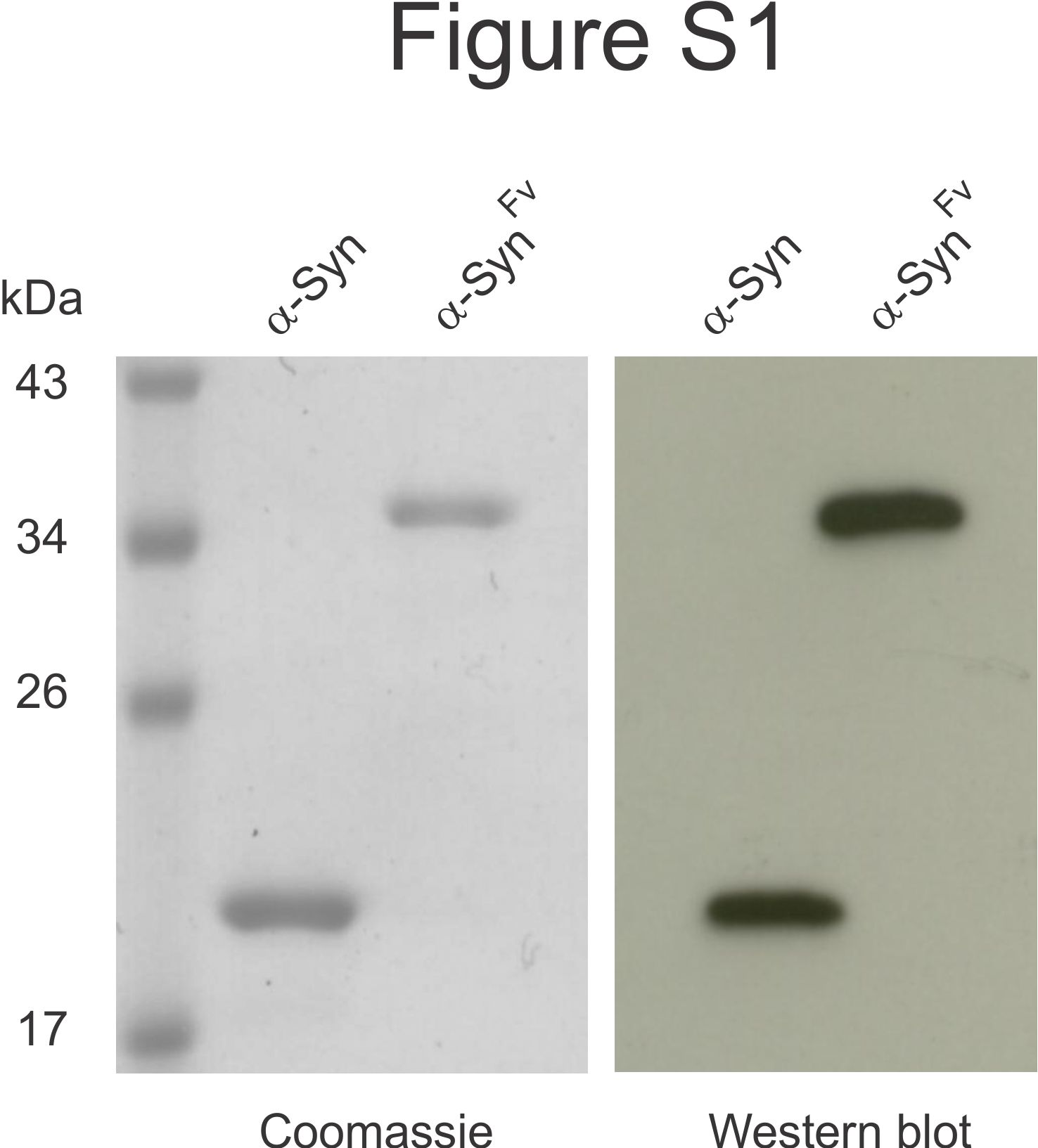

Supplement: Additional file 1: Figure S1 — Expression and purification of α-Syn and α-SynFv. Recombinant proteins were produced in BL21(DE3)pLysS Escherichia coli cells, as described under “Materials and Methods”. 2 μg of recombinant proteins was subjected to the SDS-PAGE and Coomassie blue staining (left panel). Purity of the proteins was estimated to be superior to 95%. Identity of the proteins was confirmed by immunoblotting using monoclonal anti-α-Syn EP1646Y antibody (right panel). [file 1750-1326-8-5-S1.jpeg]

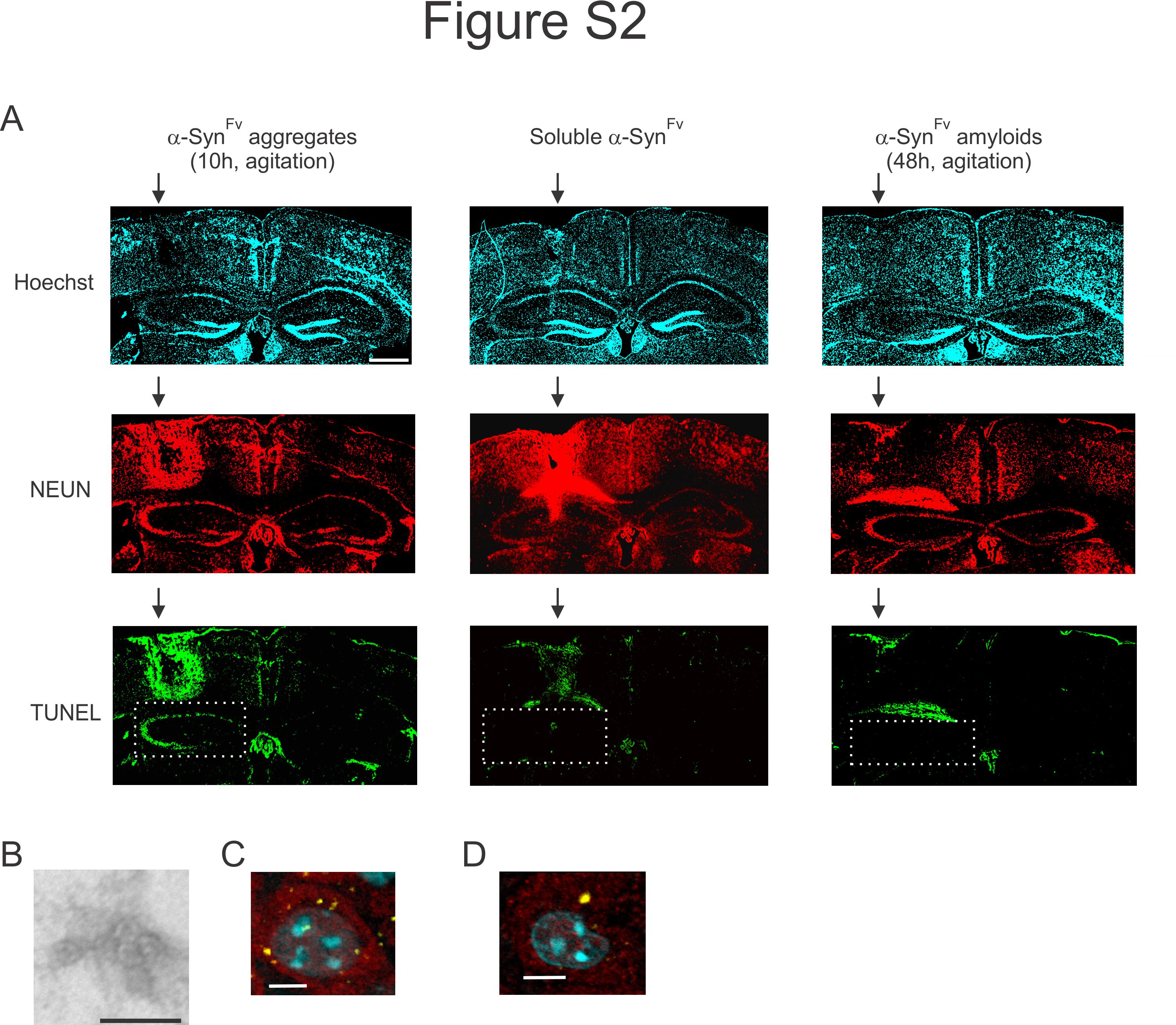

Supplement: Additional file 2: Figure S2 — In vivo neurotoxicity of synthetic α-SynFv oligomers. (A) α-SynFv protein was treated in the absence (soluble) or in the presence of 10 μM AP20187 (aggregates and amyloids) as indicated, and injected into the brain of wild-type C57BL/6 mice. Apoptotic neurons of the hippocampal region adjacent to the injection site were detected by TUNEL assay (green channel) followed by confocal microscopy. Hippocampus in the injected hemisphere is shown in a dotted box. The neuronal marker NeuN is detected by immunofluorescence (red channel). Nuclei are stained with Hoechst (blue channel). Scale bar, 500 μm. (B) Electron micrographs of toxic α-SynFv oligomers obtained after incubation at 37 ºC for 10 h. Scale bar, 25 nm. (C) Synthetic α-SynFv aggregates were labeled with Alexa Fluor 633 and detected by confocal microscopy (aggregates, yellow channel; NeuN, red channel; Nucleus, Blue channel). Scale bar, 10 μm. (D) Synthetic α-SynFv amyloids were labeled with Alexa Fluor 633 and detected by confocal microscopy (aggregates, yellow channel; NeuN, red channel; nucleus, blue channel). Scale bar, 10 μm. [file 1750-1326-8-5-S2.jpeg]

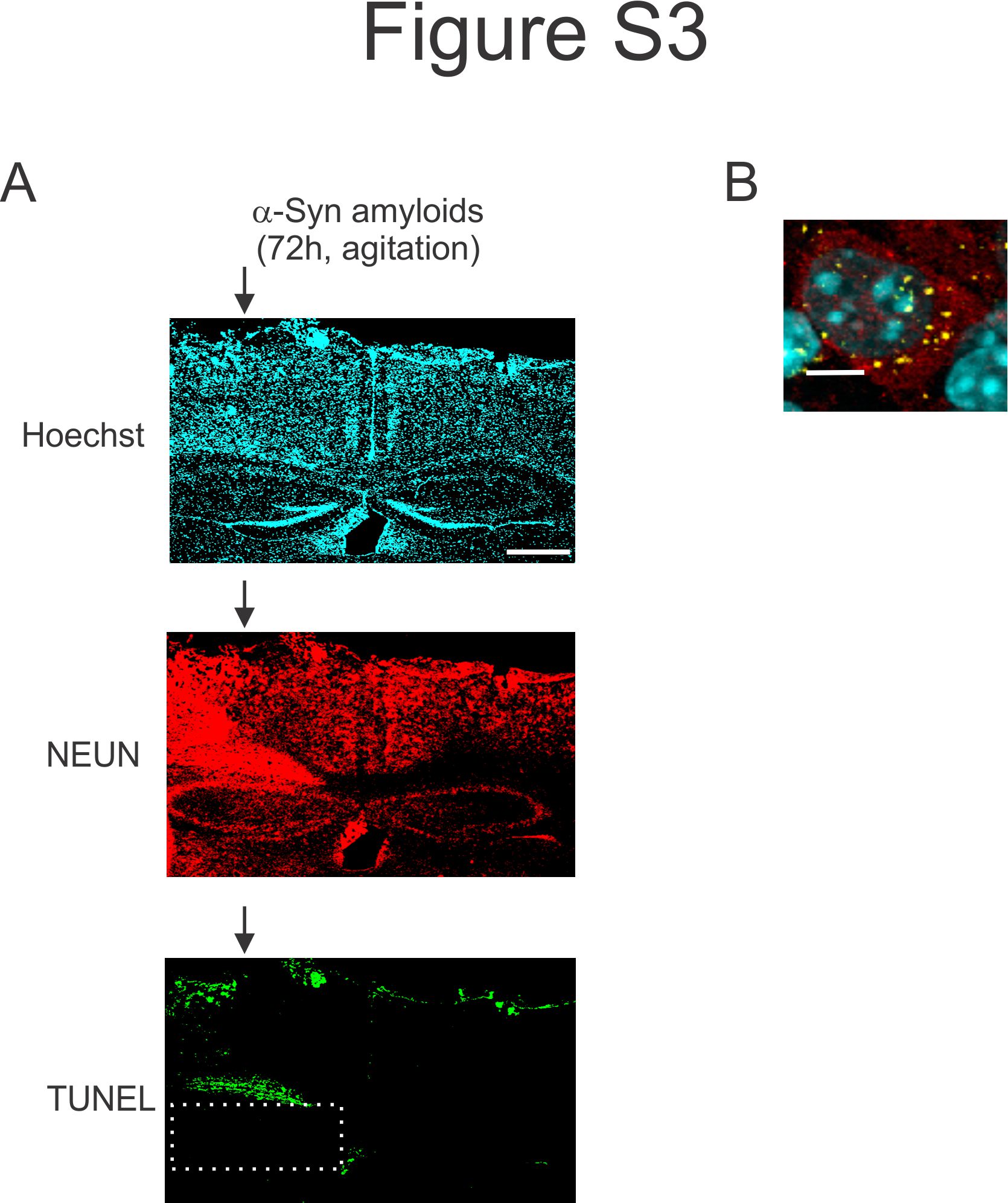

Supplement: Additional file 3: Figure S3 — Absence of in vivo neurotoxicity of synthetic α-Syn amyloids. (A) α-Syn protein was treated as indicated, and injected into the brain of wild-type C57BL/6 mice. Apoptotic neurons of the hippocampal region adjacent to the injection site were detected by TUNEL assay (green channel) followed by confocal microscopy. Hippocampus in the injected hemisphere is shown in a dashed box. The neuronal marker NeuN is detected by immunofluorescence (red channel). Nuclei are stained with Hoechst (blue channel). Scale bar, 500 μm. (B) Synthetic α-Syn amyloids were labeled with Alexa Fluor 633 and detected by confocal microscopy (aggregates, yellow channel; NeuN, red channel; nucleus, blue channel). Scale bar, 10 μm. [file 1750-1326-8-5-S3.jpeg]

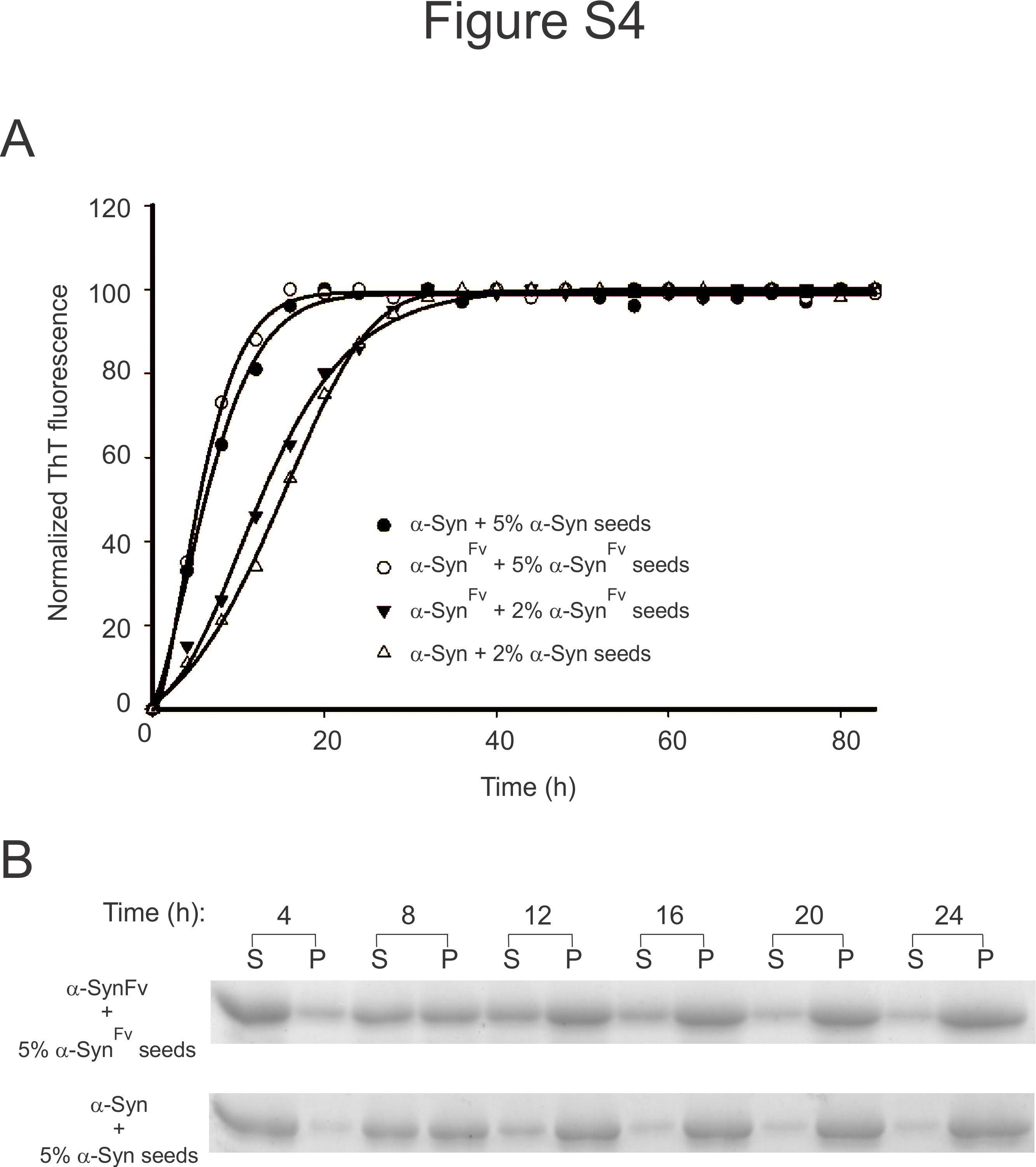

Supplement: Additional file 4: Figure S4 — Acceleration of α-Syn amyloid formation in seeding incubations. (A) 200 μM of freshly prepared recombinant α-Syn or α-SynFv was incubated with 2% or 5% homologous amyloid seeds with agitation at 37°C for 80 h. α-SynFv seeds were produced in the presence of AP20187. α-Syn seeds were obtained by agitating α-Syn for 48 h at 37ºC. (B) Aliquots withdrawn at 4 h intervals from the incubations including 5% seed were analyzed by centrifugation, SDS-PAGE and Coomassie blue staining. [file 1750-1326-8-5-S4.jpeg]

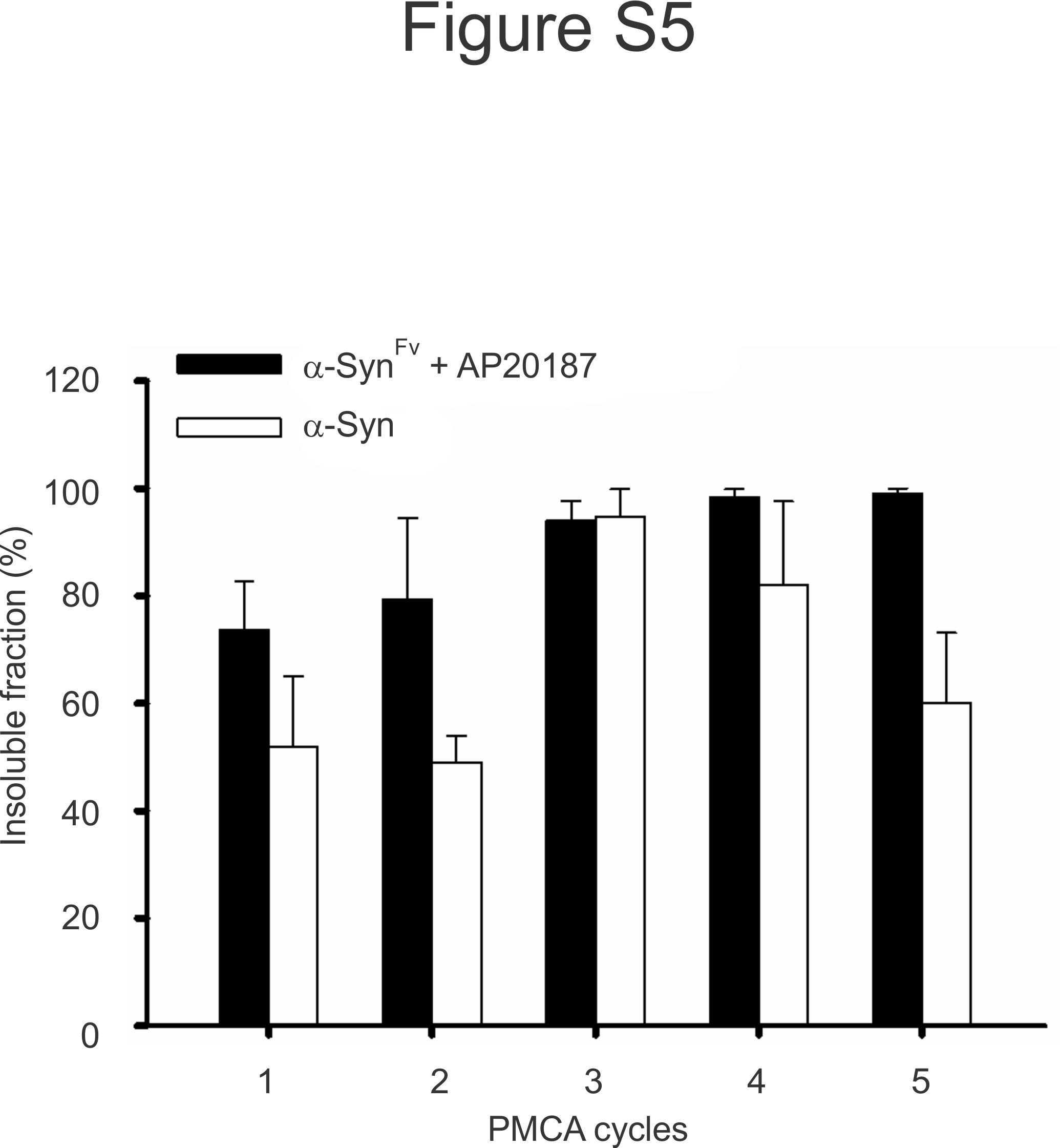

Supplement: Additional file 5: Figure S5 — Densitometric analysis of PMCA reactions with α-Syn and α-SynFv in three independent experiments. α-Syn aggregates or α-SynFv aggregates induced by AP20187 were diluted 20 times into freshly prepared soluble proteins of the same species. Supernatant (S, soluble) and pellet (P, insoluble) fractions recovered from centrifugation of 3 μg protein samples withdrawn at the end of each PMCA cycle were analyzed by SDS-PAGE and Coomassie blue staining. [file 1750-1326-8-5-S5.jpeg]

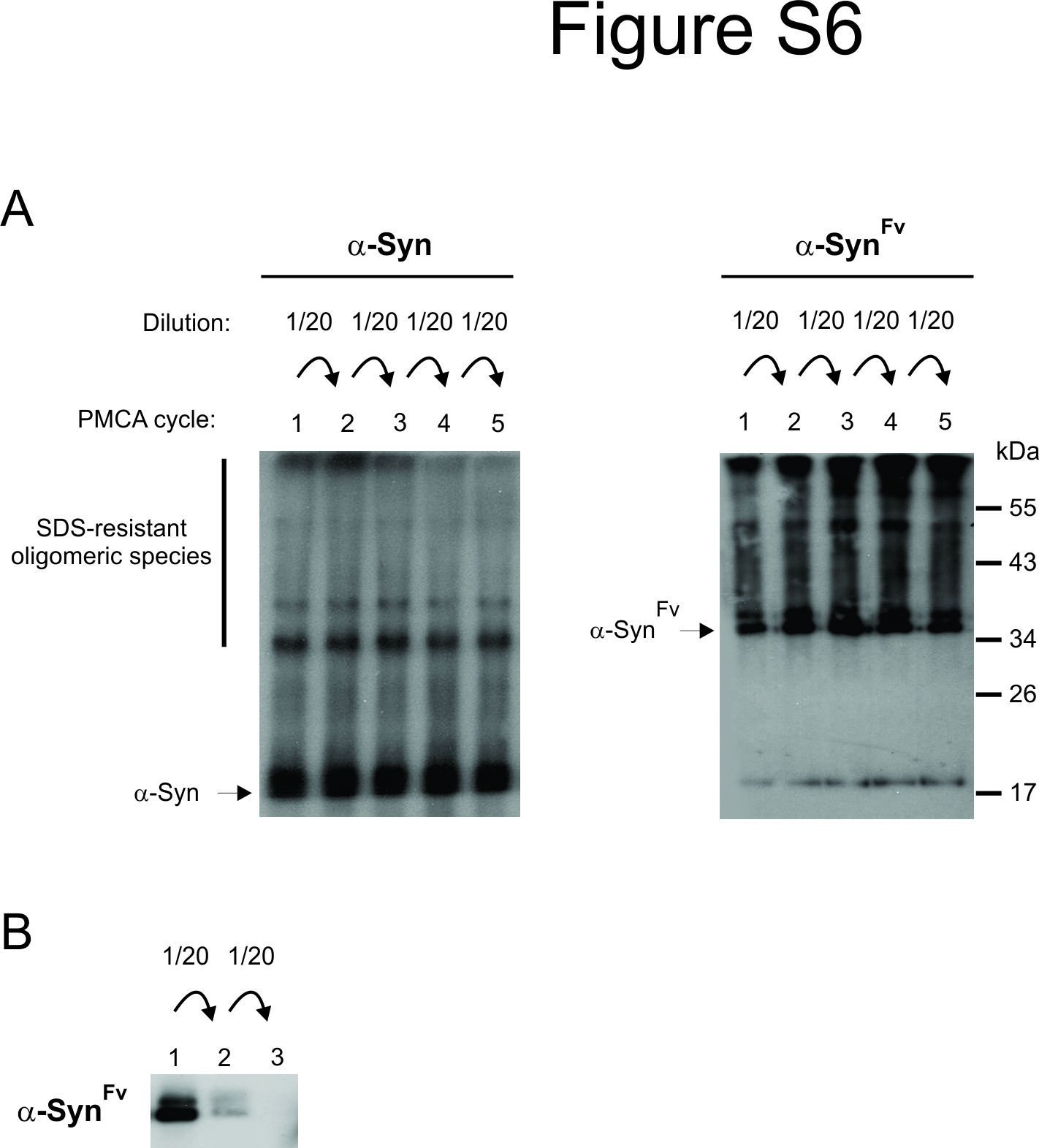

Supplement: Additional file 6: Figure S6 — Analyses of PMCA reactions. (A) SDS-resistant α-Syn and α-SynFv species after PMCA in pellet fractions. At the end of each PMCA cycles using purified recombinant proteins (Figure Figure 5), proteins in the pellet were analyzed by western blot using monoclonal anti-α-Syn EP1646Y antibody. (B) Control experiments in the absence of seeds. The PMCA reaction was not seeded with α-SynFv aggregates. At the end of each PMCA cycle, proteins in the pellet were analyzed by western blot using monoclonal anti-α-Syn EP1646Y antibody. [file 1750-1326-8-5-S6.jpeg]
