# Supplementary material for: Aggregation and neurotoxicity of recombinant α-synuclein aggregates initiated by dimerization
Source: Mol Neurodegener. 2013 Jan 22;8:5. doi: 10.1186/1750-1326-8-5 (PMC3764494; doi:10.1186/1750-1326-8-5)
Supplement: Additional file 7: Table S1 — Oligonucleotides used to produce α α-Syn constructs. [file 1750-1326-8-5-S7.pdf]

Table S1. Oligonucleotides used to produce α-Syn constructs

Oligonucleotide Name	Sequence (5'-3')
α-Syn-Fw	CTTGCCTTCAAGCCTTCTGCCTTT
α-Syn-Rv	GGCACATTGGAACTGAGCACTTGT
α-Syn-His-Fw	GCCGGCATCATATGGATGTATTCATGAAAGG
α-Syn-His-Rv	ATGCAAGCTTAGTGGTGGTGGTGGTGGTGGGCTTCA GGTTCGTAGTC
α-Syn-Fv-Fw	GACTACGAACCTGAAGCCTCTAGAGGAGTGCAGGTG
α-Syn-Fv-Rv	CACCTGCACTCCTCTAGAGGCTTCAGGTTCGTAGTC
Fv-His-Rv	ATGCAAGCTTAGTGGTGGTGGTGGTGGTGTGCGTAG TCTGGTACGTCG
α-Syn-Fw$^{Y136\text{-}TAT}$	GGAAGGGTATCAAGACTATGAACCTGAAGCCCACC
α-Syn-Rv$^{Y136\text{-}TAT}$	GGTGGGCTTCAGGTTCATAGTCTTGATACCCTTCC
α-Syn-Fv-Fw$^{Y136\text{-}TAT}$	GGAAGGGTATCAAGACTATGAACCTGAAGCCTCTAG AGG
α-Syn-Fv-Rv$^{Y136\text{-}TAT}$	CCTCTAGAGGCTTCAGGTTCATAGTCTTGATACCCTT CC
